# Permissivity of the NCI-60 cancer cell lines to oncolytic Vaccinia Virus GLV-1h68

**DOI:** 10.1186/1471-2407-11-451

**Published:** 2011-10-19

**Authors:** Maria Libera Ascierto, Andrea Worschech, Zhiya Yu, Sharon Adams, Jennifer Reinboth, Nanhai G Chen, Zoltan Pos, Rahul Roychoudhuri, Giovanni Di Pasquale, Davide Bedognetti, Lorenzo Uccellini, Fabio Rossano, Paolo A Ascierto, David F Stroncek, Nicholas P Restifo, Ena Wang, Aladar A Szalay, Francesco M Marincola

**Affiliations:** 1 Infectious Disease and Immunogenetics Section (IDIS), Department of Transfusion Medicine, Clinical Center and trans-NIH Center of Human Immunology (CHI), National Institutes of Health, Bethesda, MD, USA; 2 Department of Internal Medicine and Center of Excellence for Biomedical Research (CEBR), University of Genoa, Italy; 3 Genelux Corporation, San Diego Science Center, San Diego, CA, USA; 4 Surgery Branch, National Cancer Institute, Bethesda, MD, USA; 5 Warren G. Magnuson Clinical Center, HLA Laboratory, National Institutes of Health, Bethesda, MD, USA; 6 Department of Internal Medicine II Julius-Maximilian University of Würzburg, Würzburg, Germany; 7 Department of Biochemistry, Biocenter, University of Würzburg, D-97074 Würzburg, Germany; 8 NIDCR/Molecular Physiology and Therapeutics Branch, National Institutes of Health, Bethesda, MD, USA; 9 Department of Molecular Pathology, University Federico II, Via G. Pansini, Naples, Italy; 10 National Cancer Institute, "Fondazione G Pascale", via G. Semmola, Naples, Italy; 11 Cell Therapy Section, Department of Transfusion Medicine, Clinical Center, National Institutes of Health, Bethesda, MD, USA; 12 Department of Oncology, Biology and Genetics and Department of Internal Medicine, University of Genoa, Italy; 13 Department of Medical Oncology, National Cancer Research Institute, Genoa, Italy; 14 Institute of Infectious and Tropical Diseases, University of Milan, L. Sacco Hospital, Milan, Italy; 15 Department of Radiation Oncology, Rebecca & John Moores Comprehensive Cancer Center, University of California, San Diego, CA, USA

## Abstract

**Background:**

Oncolytic viral therapy represents an alternative therapeutic strategy for the treatment of cancer. We previously described GLV-1h68, a modified Vaccinia Virus with exclusive tropism for tumor cells, and we observed a cell line-specific relationship between the ability of GLV-1h68 to replicate in vitro and its ability to colonize and eliminate tumor in vivo.

**Methods:**

In the current study we surveyed the in vitro permissivity to GLV-1h68 replication of the NCI-60 panel of cell lines. Selected cell lines were also tested for permissivity to another Vaccinia Virus and a vesicular stomatitis virus (VSV) strain. In order to identify correlates of permissity to viral infection, we measured transcriptional profiles of the cell lines prior infection.

**Results:**

We observed highly heterogeneous permissivity to VACV infection amongst the cell lines. The heterogeneity of permissivity was independent of tissue with the exception of B cell derivation. Cell lines were also tested for permissivity to another Vaccinia Virus and a vesicular stomatitis virus (VSV) strain and a significant correlation was found suggesting a common permissive phenotype. While no clear transcriptional pattern could be identified as predictor of permissivity to infection, some associations were observed suggesting multifactorial basis permissivity to viral infection.

**Conclusions:**

Our findings have implications for the design of oncolytic therapies for cancer and offer insights into the nature of permissivity of tumor cells to viral infection.

## Background

Despite improvements in conventional cancer treatment the prevalence of cancer-related deaths has minimally changed and novel therapeutic strategies are continuously sought. Among them, replication competent oncolytic viruses are increasingly studied because of their intrinsic tumor tropism [1]. This tropism for cancer cells is engineered by the disruption of non-essential viral genes altering their replicative capacity in a tissue-specific fashion [2-4].

Their selective intra-tumoral replication leads to killing of infected cancer cells by virus-specific and apoptosis-independent mechanisms (oncolysis) [5] or through activation of anti-viral immune mechanisms that clear tumors cells infected with virus [6].

Among poxviruses, Vaccinia Virus (VACV) is a promising candidate for oncolytic therapy due to its extensive past utilization for worldwide vaccination against smallpox that demonstrated its safety in humans.

Although VACV is known to infect a variety of mammalian cell lines, recent evidence highlights the importance of host restriction in infection permissivity. Genes known to influence the ability of VACV to infect cells, termed host range genes, have been identified, and hypothesized to block productive infection at different points in the replication cycle [7,8]. In addition to host genes, a number of other factors might influence the permissivity to infection of a given cell type, such as its tissue accessibility *in vivo*, the special vascular structure of tumor, the density of cellular receptors for the virus, the ability to internalize the virus, the metabolic state of the cell and intrinsic genetic differences within each viral species [8]. Thus, we hypothesized that, although receptors for VACV are believed to be ubiquitous, and VACV replication is relatively independent from the host cell, virus tropism may be determined by other subtle factors that may be dependent upon the cell type and its metabolic state; the same factors may affect the efficiency of replication in different cancers *in vitro *and/or *in vivo*.

Recently, Zhang *et al *introduced GLV-1h68 as a recombinant VACV derived from LIVP wild-type strain by insertion of three expression cassettes encoding *Renilla *luciferase-*Aequorea *green fluorescent protein fusion (Ruc-GFP), beta-galactosidase (β-gal) and beta-glucuronidase (β-glu) into the *F14.5L, J2R *(encoding thymidine kinase) and *A56R *(encoding haemagglutinin) respectively [7]. The ability to replicate exclusively within tumors while completely sparing non malignant tissues, makes GLV-1h68 systemic administration a promising tool capable of safely eradicating pancreatic cancer malignant pleural mesothelioma [9], breast carcinoma [7], anaplastic thyroid cancer [10] and squamous cell carcinoma xenografts [11]. In a xenograft model, we recently observed a correlation between the cell line-specific ability of GLV-1h68 to replicate *in vitro *in the first 20 hours of infection and its effectiveness *in vivo *in colonizing and causing regression of the corresponding tumor implants [12]. Thus, we screened a panel of cell lines, often used as a standard for the study of cancer therapeutics, for their permissivity to VACV infection/replication *in vitro*. Our final intent was developing a roadmap for the design and interpretation of future studies adopting viral oncolytic strategies. We screened the NCI-60 panel constituted by cancer cell lines of diverse lineage derived from nine distinct tissues (breast, colon, central nervous system, renal, lung, melanoma, ovarian, prostate, and blood) previously characterized extensively by the National Cancer Institute [13-17]. In addition, we screened 15 cell lines that we previously characterized for their *in vitro *permissivity to GLV-1h68 replication as well as *in vivo *responsiveness to GLV-1h68 oncolytic effects [12]. Infection with other non-receptor dependent viruses such as Vesicular Stomatitis Virus (VSV) and wild-type strain Western Reserve (WR) Vaccinia Virus were performed on several cell lines to test whether information obtained studying GLV-1h68 could be generalized to other viral constructs. All cell lines were grown and tested in identical conditions and their transcriptional patterns before infection (as a predictor of permissivity to infection) were compared. We observed that permissivity to VACV replication in the first 20 hours following infection is heterogeneous among cell lines but highly reproducible within each cell line. The tissue of origin of each cell line does not influence permissivity to infection with the exception of B cell lymphomas. Permissivity was similar between two independent VACV constructs and between them and VSV suggesting that a general characteristic of the cancer cell may be a common determinant for the replication of these viruses. Finally, although a single transcriptional signature predictive of cell line permissivity was not identified, several associations were found suggesting a multifactorial control of viral replication in this model system.

## Methods

### Cancer cell lines

Fifty-nine available cell lines from the NCI-60 panel were purchased from NCI-Frederick Cancer Center DCTD Tumor/Cell Repository. GI-101A cells were kindly provided by Dr. A. Aller, Rumbaugh-Goodwin Institute for Cancer Research, Inc., Plantation, Florida and Huh7.5.1 by Dr. Richard Wang, Department of Transfusion Medicine, NIH, Bethesda, MD. Three autologous melanoma cell lines (888-MEL, 1858-MEL and 1936-MEL) from temporally distinct cutaneous metastases were derived as previously described [18,19]. MIAPaCa2, HT29, A549, OVCAR3, Panc-1, Siha, MDA-MB-231, NCI-H1299 and PC-3 were purchased from American Type Culture Collection (Manassas) in the past and have been previously characterized for their *in vivo *responsiveness to viral oncolytic therapy with GLV-1h68 by our group [12].

All cells were cultured in Roswell Park Memorial Institute Medium (RPMI) supplemented with 10% FBS, 10 mM HEPES, 1% antibiotic/antimycotic solution. All cell cultures were carried out at 37°C under 5% CO_2_. During prolonged cell culture and immediately before DNA, RNA isolations and viral infection, all cells were tested for mycoplasma with the Venor^®^GeM Mycoplasma Detection kit (Sigma, St Louis, MO). In no occasion contamination was detected. Original HLA class I and II loci sequence-based typing was performed on the NCI-60 as previously described [20].

### Viral constructs and infections

The construction of mutant VACV GLV-1h68 was described previously [7]. Vaccinia Virus WR-GFP and VSV-GFP were kindly provided respectively by Drs. N. Restifo, NCI, Bethesda, MD and S. Balachandran, Fox Chase Cancer Center, Philadelphia, PA [21]. Before carrying out the experimental procedure, cells were thawed and cultured for three days in RPMI supplemented with antibiotics. One day following the beginning of culture cells were harvested for DNA and RNA isolation while a separate aliquot was exposed to viral infection. For DNA and RNA isolation, 2 × 10^6 ^cells were used and 5 × 10^5 ^cell were used for viral infection.

Cells were infected with VACV GLV-1h68, at multiplicity of infection (MOIs) of 0.3 and 0.6 or with Vaccinia WR and VSV virus using MOI at 10 and 0.002 respectively. After infection the cells were incubated in cell culture medium at 37°C for 20 hrs. One hour after infection the culture media was replaced with fresh media and cells were incubated at 37°C for 20 hrs.

### Quantitative real-time PCR validation of VACV 1h68 gene expression

Differential expression of viral genes in infected cells was detected by using TaqMan^® ^Gene Expression Assays (Applied Biosystems, Foster City, CA). RNA was reverse transcribed as above with random oligo primers in 20 μl final volume. Primer Express 2 (PE2) (Applied Biosystems, Foster City, CA) was used to generate primers and a TaqMan probe specific for the virus sequence; real-time PCR was performed on thermal cycler 7900HT (Applied Biosystems). Differences in expression were determined by the ratio of Ct values of viral genes over those of endogenous control (18s rRNA).

### Plaque forming assay

A375, DU-145 and A549 cell lines were infected with GLV-1h68 at an MOI of 0.6. At 8, 20 and 36 hours after infection, tumor cells were harvested and lysates were prepared by freeze-thawing and sonication in 1 ml of RPMI media with 10% FBS. The viral titers were determined by plaque forming assay using TK-cells. TK-cells (kindly provided by Dr. Jack Bennink, NIAID Bethesda, MD) were cultured in DMEM media with 10% FBS in 6 well plates. When cell density reached 80%-90% confluence, they were washed with 0.1% BSA in PBS and infected with 0.5 ml of diluted cell lysate in PBS with 0.1% BSA for 2 hours at 37°C incubator. Two ml of cell culture media were then added to each well and incubated at 37°C and 5% CO2 for 48 hours. Media were removed from the cells culture and stained with crystal violet (Sigma, St. Luis, MO) and washed with PBS. The titer of the virus was determined by the plaque numbers of the highest dilution. Each cell lysate were titered in duplicate.

### FACS-analysis of GFP Protein expression

Twenty hours following viral infection, cancer cells were trypsinized (when applicable), fixed by paraformaldehyde, washed with AUTOMacs running buffer and GFP expression was analyzed with a FACSCalibur flow cytometer. Data were analyzed by the FlowJo software. To ensure between-batch reproducibility, all FACS experiments included BD's Rainbow Calibration Particles (6 peaks) as standards and measured signal intensities were converted in molecules of equivalent fluorescein, phycoerythrin, or allophycocyanin (MEFL, MEPE, MEAPC). The flow cytometer settings were kept constant during the complete experimental procedure and the cell line A549 was included in all experimental batches as a positive control for viral infection to asses inters experimental consistency.

### Nucleic acid isolation and preparation

Automated DNA isolation was performed from non-infected human cancer cell lines using Fujifilm's Quickgen DNA Whole Blood kit and Nucleic Acid Isolation System-810. Total RNA (tRNA) from tissue cultures was isolated with the Qiagen miRNeasy Mini kit and its quality was tested with the Agilent Bioanalyzer 2000 (Agilent Technologies). For expression studies, tRNA was amplified into antisense RNA (aRNA) as previously described [22,23]. Reference for human arrays was obtained by pooling PBMCs from 4 normal donors.

### Plaque forming assay on cells cultured with specific culture media

HT29, Panc 1, NCI-H1299 and A549, cell lines were simultaneously cultured in four different cell culture media which were prepared as followed:

#### *Medium 1 *(optimized for HT-29 cells)

RPMI 1640 supplemented with 10% FBS and 1% antibiotic/antimycotic solution.

#### *Medium 2 *(optimized for Panc1)

Dulbecco's modified Eagle's medium (DMEM) supplemented with 10% (FBS) and 1% antibiotic/antimycotic solution.

#### *Medium 3 *(optimized for NCI-H1299 cells)

RPMI 1640 supplemented with 10% FBS, 4.5 g/L glucose, 10 mM HEPES, 1 mM sodium pyruvate and 1% antibiotic/antimycotic solution.

#### *Medium 4 *(optimized for A549 cells)

Kaighn's Modification of Ham's F-12 Medium (F-12K Medium) supplemented with 10% FBS and 1% antibiotic/antimycotic solution.

Cells lines were infected with GLV-1h68 at an MOI of 0.6. Twenty hours after infection, tumor cells were harvested and lysates were prepared by freeze-thawing and sonication in 1 ml of appropriate media. The viral titers were determined by plaque forming assay using TK-cells as above.

### Transcriptional analysis

Array quality was documented as previously described [24] . For 36k human array hybridization, a two color system was used; both reference and test aRNA were directly labeled using ULS aRNA Fluorescent Labeling kit (Kreatech Diagnostics, Amsterdam, The Netherlands) with Cy3 for reference and Cy5 for test samples and co-hybridized to the slides. After 20 h incubation at 42°C the arrays were washed, dried and scanning using the Agilent scanner. VACV-gene expression was assessed by a custom-made VACV array platform (VACGLa520445F, Affymetrix, CA) including 308 probes representing 219 genes that covered the combined genome of several VACV strains, the *Renilla *luciferase-*Aequorea *green fluorescent fusion gene specific for GLV-1h68, and 337 human or mouse "housekeeping" genes (393 probes) [12]. Five μg tRNA were amplified using the GeneChip^® ^One-Cycle Target Labeling and Control kit (Affymetrix, Santa Clara, CA). After 16 h incubation in the hybridization oven at 45°C, the arrays were washed and stained in the Fluidics station using the GeneChip^® ^Hybridization, Wash, and Stain Kit (Affymetrix).

### Data processing and statistical analysis

Transcriptional data were uploaded to the mAdb databank (http://nciarray.nci.nih.gov) and further analyzed using BRBArrayTools developed by the Biometric Research Branch, NCI (http://linus.nci.nih.gov/BRB-ArrayTools.html) [25], Partek Genomics Suite (St Louis, MO) or TreeView software [26]. Gene ratios were average corrected across experimental samples and displayed according to uncentered correlation algorithm. Class comparison was performed using parametric unpaired Student's t test or 3-way ANOVA. Adjustments for multiple test comparisons were based on univariate and multivariate permutation test. Gene function interpretation was based on Ingenuity Pathway Analysis (IPA, Ingenuity Systems). Data retrieved from the Affymetrix platform was normalized using median over entire array as reference because of single color labelling technology.

## Results and Discussion

### Validation of tumor cell lines

To confirm cell line identity and exclude possible culture contamination, all cell lines used in this study were typed for HLA class I which cross referenced to previous high resolution sequence based typing of NCI-60 [20] or other cell lines. Among the 74 cell lines tested, 8 (10.8%) had not been previously genotyped, 58 (78.3%) matched previous genotyping results and 8 (10.8%) displayed a different genotype and, therefore, their identity could not be verified (Table 1). In no case more than two alleles per locus were observed excluding contamination of cultures with multiple cell lines. Although the identity of 8 cell lines could not be confirmed, we used their data for this study because all analyses were performed contemporarily on the same cell lines and general information about cell line permissivity and its relationship with transcriptional data could be compared directly within each cell line independently of identity.

**Table 1:** HLA class I genotyping results of the 74 cell lines used in the study

Origin	Cell line	A Locus	B Locus	Cw Locus	Origin	Cell line	A Locus	B Locus	Cw Locus
Lung	A549	25, 30	18, 44	12, 16	Liver	Huh 7.5.1	11	54	1

	$ A549 p107	25, 30	18, 44	12, 16	Melanoma	*$ 1858-MEL	01, 24	52, 55	01, 12

	EKVX	1	37	6		#$ 1936-MEL	3	15/95, 4901	02, 07

	HOP 92	03, 24	27, 47	01, 06		397-MEL	01, 25	08, 15/95	04, 07

	# HOP62	03, 11	40, 56	01, 03		*$888-MEL	01, 24	52, 55	01, 12

	*$ NCI-H1299	24	14	8		*A375	01, 02/92	44, 57	06, 16

	NCI-H226	01, 24	07, 39	07, 12		LOX-IMVI	11, 29	07, 44	07, 16

	NCI-H23	8001	50	6		M14	11, 24	15/95, 35	03, 04

	NCI-H322M	29	44	16		SK-MEL-2	03, 26	35, 38	04, 12

	NCI-H460	24, 68	35, 51	03, 15		SK-MEL-28	11	40	3

	NCI-H522	02/92	44, 55	03, 05		#SK-MEL-5	02/92, 32	39, 44	05, 12

Cervix	*Siha	24	40	3		#UACC-257	01, 32	08, 40	07, 15

CNS	SF-268	01, 32	08, 40	02, 07		# UACC-62	02/92, 11	07, 40	03, 07

	SF-295	01, 26	07, 55	03, 07	Ovarian	IGR-OV1	24, 33	49	7

	SF-539	02/92	08, 35	04, 07		NCI/ADR-RES	01, 25	57	6

	SNB-19	02/92	18	5		OVCAR-3	02/92, 29	07, 58	7

	SNB-75	02/92, 11	35, 39	04, 12		OVCAR-3 p7	02/92, 29	07, 58	7

	U251	02/92	18	5		# OVCAR-4	02/92	18, 44	05, 07

Colon	COLO 205	01, 02/92	07, 08	7		OVCAR-5	01, 02/92	08, 44	05, 07

	HCC 2998	02/92, 24	37, 40	04, 06		OVCAR-8	01, 25	57	6

	HCT-116	01, 02/92	18, 45	05, 07		SK-OV-3	03, 68	18, 35	04, 05

	HCT-15	02/92, 24	08, 35	04, 07	Pancreas	*$ MIA Paca2	24, 32	40, 40	2

	HT-29	01, 24	35, 44	4		*$ Panc 1	02/92, 11	38	12

	$ HT-29 p155	01, 24	35, 44	4	Renal	786-0	3	07, 44	05, 07

	KM12	02/92	7	7		A498	02/92	8	7

	SW-620	02/92, 24	07, 15/95	7		ACHN	26	49	7

Breast	BT-549	01, 02/92	1517, 55	03, 07		# CAKI-1	02/92, 24	37, 40	04, 06

	*$GI-101A	29	07, 08	07, 15		RXF 393	02,24	1444	50,802

	# HS-578T	03, 11	40, 56	01, 03		SN12C	03, 24	07, 44	05, 07

	MCF-7	02/92	18, 44	5		TK-10	33	14	8

	$ MDA-MB-231 p41	02/92	40, 41	02, 17		UO-31	01, 03	07, 14	07, 08

	MDA-MB-231 p6	02/92	40, 41	02, 17	Hematopoietic	CCRF-CEM	01, 31	08, 40	03, 07

	MDA-MB-435	11, 24	15/95, 35	03, 04		HL-60	1	57	6

	T-47D	33	14	8		K-562	11, 31	18, 40	03, 05

Prostate	$ DU-145	03, 33	50, 57	6		MOLT-4	01, 25	18, 57	06, 12

	PC-3 p35	01, 24	13, 55	01, 06		RPMI 8226	30,68	15,15	02.,03

	PC-3 p7	01, 24	13, 55	01, 06		SR	02/92, 03	37, 39	06, 12

($) Cell lines previously characterized by our group for their *in vivo *responsiveness to GLV - 1h68 oncolityc therapies [12].

Among all cells lines, 58 (78.3%) matched previous genotyping results conducted in our lab, 8 (10.8%) had not been previously genotyped (*) and 8 cell lines (10.8%) displayed a different genotype from our previous genotyping (#) [20].

However, their patterns cannot be extrapolated to other homonymous cell lines of the same tissue origin at large because it was impossible to clearly identify and classify those cell lines.

### GFP gene expression correlates with viral transcription

**Figure 1:**
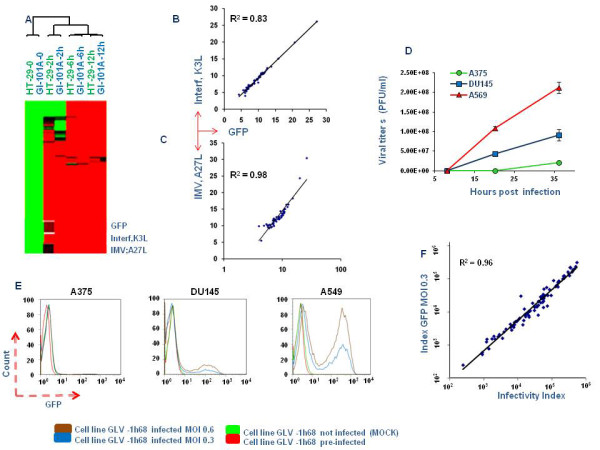
**Correlation of GFP gene expression with viral transcription, viral copy number and protein expression in cell lines infected with GLV-1h68**. A) HT-29 and GI-101A cells infected with GLV-1h68 at different time points and hybridized to a customized whole genome Vaccinia Virus chip; B, C) Screening by RT-PCR of GFP, IMV and Interf proteins in 46 cell lines infected with GLV-1h68. D) Plaque forming assay of A375, DU-145 and A549 cell lines infected with GLV-1h68 for 8, 20 and 36 hours. E) FACS analysis of GFP expression in 74 cell lines (figure showed only 3 of them) after infection of with GLV-1h68. Infections with GLV-1h68 were performed using multiplicity of infections (MOI) 0.3 and MOI 0.6; an almost perfect correlation (R^2 ^= 0.96) between the infectivity index obtained in the two conditions (F).

To test whether green fluorescent protein (GFP) expression by GLV-1h68 correlated with the global transcriptional activity of GLV-1h68 within infected cells, we hybridized RNA from HT-29 and GI-101A cells infected with GLV-1h68 at different time points to a customized whole genome VACV chip and we monitored the expression of viral genes including Ruc-GFP-fusion. GFP expression was highly correlated with the expression of the remaining 219 genes included in the chip. In particular, GFP expression occurred in the intermediate-late phase of viral genome expression following the expression of early-intermediate genes (like Interferon resistant protein or Interf) and together with other intermediate-late (IMV surface protein or IMV) genes (Figure 1A). Moreover, to confirm these finding, we infected 46 cell lines with GLV-1h68 for 20 hours and tested the expression of GFP and the two viral nonstructural proteins Interf (or K3L) and IMV (or A27L) by RT-PCR. A tight correlation was observed between the expression of GFP and Interf (R^2 ^= 0.83) or GFP and IMV (R^2 ^= 0.96), (Figure 1B, C). Since the transcriptional expression of GFP is observed in intermediate-late phase and correlated well with the expression of other early or late vaccinia genes at the time point studied, we concluded that GFP is a reliable index of active intra-cellular virally-driven transcription and, indirectly, represent a marker of viral replication.

### GFP gene expression correlates with viral copy number estimated by plaque forming assay

To investigate whether GFP positive cells harbor replicating viral particles, we titered the number of plaque forming unit in lysate of infected cells and correlated with the expression level of GFP.

A375, DU-145 and A549 cell lines (showing respectively low, intermediate or high GFP by RT-PCR) were infected with GLV-1h68 for 8, 20 and 36 hours. At the indicated time points, infected cells were collected and viral titers were extrapolated in a plaque assay using TK-cell lines. A375 showed the lowest permissivity to VACV compared to DU-145 and A549 confirming a correlation with RT-PCR based estimates of GFP expression (R^2 ^= 0.76) (Figure 1D).

### GFP gene transcription correlates with its protein expression

**Figure 2:**
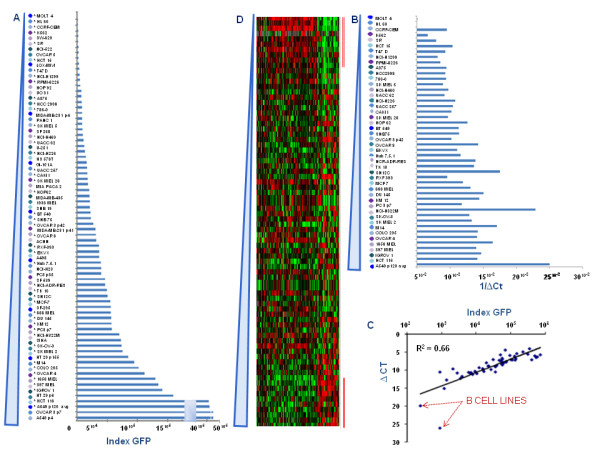
**Expression of GFP in 74 cell lines infected with GLV-1h68**. A) GFP protein expression data, ranked among the 74 cell lines according to infectivity index. With * are indicated the 46 cells previously analyzed by RT-PCR. B) Side by side comparison of 1/ΔCt (1/Ct GFP - Ct 18S) levels obtained by RT-PCR with GFP expression levels obtained by FACS showed similar pattern which is also highlighted by the scatter plot comparing GFP protein and RNA levels (R^2 ^= 0.66) (C). D) Heat map of the 371 genes, identified by Student's *t *test (p 0.01) comparing 10 highest and 10 lowest GLV 1h68 permissive cell lines. The cells used for the comparison are indicated with a red bar.

To determine whether the GFP gene transcription correlates with its protein expression, seventy-four cancer cell lines were infected with GLV-1h68 and GFP expression was evaluated by FACS analysis. The infection of the 74 cell lines was performed in batches of five; A549, evaluated to be highly permissive to GLV-1h68 replication RT-PCR, was repeatedly used as a positive control and as cross-reference among different batches. The cell line demonstrated a highly comparable behaviour in each batch (data not shown). Since different cell lines were characterized by different auto-fluorescence, GFP intensity of uninfected cells was subtracted from GFP intensity of infected cells. In addition, cells were observed to vary not only for fluorescence of intensity (MFI) within the GFP positive cells but also for the percentage of fluorescent cells. Therefore for each cell line we calculated the infectivity index as the product the two parameters (MFI of GFP and the percentage of fluorescent cells). Although this index was used for subsequent analyses, either MFI or percent of infection tightly correlated with the parameter and the salient conclusions would not change based on the adoption significantly. We observed a continuous spectrum of GFP expression among cell lines (Figure 1E, Table 2). Infections were performed using multiplicity of infections (MOIs) 0.3 and MOI 0.6. An almost perfect correlation (R^2 ^= 0.96) between the infectivity index obtained in the two conditions was observed with the higher MOI consistently yielding a 2-fold higher intensity (Figure 1F). This suggests that concentrations of virus used to test cellular permissivity were within a flexible dynamic range. Since results obtained with the two different MOIs were almost identical, the subsequent discussion will be focused on MOI 0.6 data even though same conclusions could be drawn using the other MOI. Moreover, a good correlation (R^2 ^= 0.66) was observed between GFP protein and RNA levels (Figure 2C) which was more significant when two outlier leukemia cell lines (HL-60 and MOLT4) completely resistant to infection were removed from the analysis (R^2 ^= 0.75).

**Table 2:** 74 cell lines ordered based on the protein level of Ruc-GFP derived by FACS Data (infectivity index)

Cell line	Ruc-GFP Index	1/Δ Ct	Cell line	Ruc-GFP Index	1/Δ Ct
*MOLT4	242.25	0.05	SNB75	22849.59	0.114

*HL-60	890.01	0.038	OVCAR-3 p42	26919.33	0.102

*CCRF-CEM	890.69	0.095	MDA-MB231 p41	30471.13	#

*K562	1185.59	0.066	OVCAR-8	31066.5	0.142

*SW-620	1223.86	#	ACHN	31639.56	#

*SR	1389.99	0.079	RXF-393	35969.66	0.095

*NCI-H522	1723.7	0.081	EKVX	36117.84	0.111

*OVCAR5	1961.62	#	A498	36486.43	#

*HCT15	2121.68	0.104	Huh 7.5.1	39321.66	0.116

*LOX-IMVI	2591.66	#	NCI-H23	40056.95	#

T47D	2831.5	0.093	PC3 p35	40124.72	#

NCI-H1299	3499.99	#	SF539	46092.96	#

RPMI-8226	3891.89	0.085	NCI-ADR_RES	46569.81	0.138

HOP92	4430.72	#	TK-10	49256.78	0.136

UO-31	5960.36	#	SN12C	50505.78	0.175

A375	6049.59	0.094	MCF-7	55230.93	0.119

HCC2998	6537.28	0.093	SF 295	56037.99	#

786-0	7141.29	0.094	888-MEL	56437.28	0.131

MDA-MB 231	7500.93	#	DU-145	56733.35	0.15

Panc1	7876.6	#	KM12	57112.82	0.144

SK-Mel 5	7917.7	0.089	PC3 p7	57919.09	0.118

SF268	8703.17	#	NCI-H322M	69687.9	0.229

NCI-H460	9247.41	0.097	Siha	72182.24	#

UACC 62	10437.13	0.092	SK-OV-3	73220.28	0.129

U-251	11245.39	#	SK-MEL2	75407.23	0.133

NCI-H226	12182.98	0.108	HT29 p155	84833.55	#

Hs578T	13912.24	#	M14	94724.62	0.171

GI-101A	14791.4	#	*Colo 205	101576.11	0.142

UACC 257	15405.44	0.105	*OVCAR-4	111530.36	0.141

Caki I	15869.9	0.102	*1858-MEL	129262.97	0.164

SK-Mel 28	16737.85	0.097	*397-MEL	134615.99	0.139

MIA PaCa2	17376.47	#	*IGR-OVI I	141059.48	0.147

HOP62	19768.46	0.126	*HT29 p6	159709.89	#

MDA-MB435	20698.92	#	*HCT 116	406258.9	0.141

1936-MEL	21079.94	#	*A549 p120 avg	409912.11	0.25

SNB19	21349.31	#	*OVCAR-3 p7	425559.88	#

BT549	22182.84	0.113	*A549 p4	425962.87	#

To test the correlation between protein and mRNA levels, expression of Ruc-GFP was screened by RT-PCR in 46 cell lines post-infected. RT-PCR values are expressed as 1/ΔCt (Ct GFP-Ct 18S). Cells further used in microarray class comparison and class prediction analysis are indicated with *.

GFP protein expression data were ranked among the 74 cell lines according to infectivity index (Figure 2A). Six out of 6 cancer cell lines of haematopoetic derivation were completely resistant to GLV-1h68 infections. The permissivity to GLV-1h68 based on GFP intensity was not affected by cells size. In fact, cells with similar size such as SK-MEL-2, and SK-MEL-5 (both very large) or A375 and M14 (both very small), showed different permissivity to viral infection. Moreover, permissivity to GLV-1h68 replication was not tissue-specific nor patient-specific as three autologous melanoma cell lines derived from the same progenitor cell clone but from temporarily distant metastases (Mel-888, MEL 1858 and MEL 1936) [18] displayed discordant behavior. Side by side comparison of 1/ΔCt expression levels according to RT-PCR (Figure 2B) demonstrated a pattern of expression similar to the infectivity index calculated according to GFP expression levels.

Detection of cells infected with GFP-carrying vaccinia provides a fast and sensitive method to measure virus replication [27] that in our hands correlated well with transcriptional expression of other VACV genes as well as the commonly used but more laborious plaque forming assay that yields information about viral particle production following cellular infection.

### Indexes are similar in other vaccinia strain and Vesicular Stomatitis Virus infected cells

In order to test whether differential replication of GLV-1h68 in various cell line is virus-specific, 6 cell lines were infected in parallel with GLV-1h68 or a different Vaccinia strain (VACV-WR) and 4 cell lines with GLV-1h68 or Vesicular Stomatitis Virus (VSV) [21], both expressing the GFP gene. Twenty hours after infection, infectivity index was calculated (Additional File 1A) revealing a good correlation in cell-specific infectivity driven by the three viruses (R^2 ^= 0.84 and R^2 ^= 0.87, comparing GLV-1h68 to VACV-WR or VSV respectively) suggesting that a common cancer cell phenotype may influence the replication of these viruses.

### Usage of specific cell culture media does not affect permissivity to GLV-1h68 infections

Since the main study was performed in standardized conditions using identical but arbitrarily selected culture media for all cell lines, we tested whether the usage of different cell cultured media could have impact on cells permissivity to GLV-1 h 68 infections. A549, HT29, Panc1 and NCI-H1299 cell lines (showing high, intermediate and low GFP by FACS analysis) were simultaneously cultured in cell specific culture media (as originally recommended by the providing sources) and infected with GLV-1h68. Twenty hours after infection cells were collected and viral titers were extrapolated in a plaque assay using TK-cell lines.

NCI-H1299 and Panc 1 showed lowest permissivity to VACV compared to HT-29 and A549. These observations confirmed the results obtained by FACS analysis and ruled out variation in permissivity to VACV infection due to cell culture conditions (Additional File 1B).

### Transcriptional profiles do not differ among cancer cell lines with different permissiveness to GLV-1h68

In order to identify correlates of permissivity to viral infection mediated by GLV-1h68, we performed transcriptional profiles of 74 cancer cell lines prior infection. The 74 mycoplasma-free cell lines used in this study were cultured in identical conditions and subjected for gene expression profiling analysis using 36 K oligo human array. The complete dataset, was filtered (80% gene presence across all experiments) to enrich for informative transcripts obtaining a total of 32,863 transcripts.

Student's t test (cut off p_2 _value ≤ 0.01) applied to the filtered data set was used to compare the 10 most to the 10 least permissive cell lines based on infectivity index (Table 2). This analysis identified 371 genes differentially expressed between the two groups (global permutation p value = 0.01). Among them, 117 were up regulated and 254 down regulated in cell lines with higher permissivity to GLV-1h68 (Table 3). Supervised clustering according to degree of GFP expression (Figure 2D) did not demonstrate a gradual change in transcription of the same genes when the independent intermediate data set was analyzed although the two classes used for class comparison (red bars over the heat map) clearly demonstrated a different pattern. Thus, this gene selection cannot be considered independently predictive of permissivity.

**Table 3:** List of genes down-regulated (on the top) and up-regulated (on the bottom) in cells more permissive to GLV-1h68 infection

Gene	Fc	Gene	Fc	Gene	Fc	Gene	Fc	Gene	Fc
LCP1	0.18	NIPSNAP3B	0.56	FBXO4	0.63	POLD3	0.68	CEP250	0.76

TMEM194A	0.32	NKTR	0.56	KHDRBS1	0.64	GABPB1	0.68	THAP1	0.76

CORO1A	0.38	RASSF1	0.56	LOC645691	0.64	HIF3A	0.68	SFRS2IP	0.77

NUCB2	0.38	ZNF189	0.56	PGM2	0.64	MBTD1	0.68	TMCO6	0.77

ARHGAP4	0.38	ZNF33B	0.56	SGOL1	0.64	SLC38A1	0.68	ARMC8	0.77

CDCA7L	0.4	CCR2	0.56	CRAMP1L	0.64	THYN1	0.69	TRAPPC6B	0.77

40792	0.43	STAU2	0.56	ARHGAP19	0.64	SLC2A14	0.69	NSA2	0.78

ACADM	0.45	MDN1	0.56	TJAP1	0.64	BIRC6	0.69	FAU	0.78

LOC100289152	0.46	EIF4B	0.56	C14orf126	0.64	GMEB1	0.69	RBBP6	0.78

DNAH11	0.47	ZFP90	0.57	TMEM18	0.64	UBE4A	0.69	UBE2V2	0.78

FBXO5	0.47	EXOSC8	0.57	DAXX	0.64	CCDC77	0.69	VCPIP1	0.79

NCRNA00201	0.48	H2AFV	0.57	TUBB	0.64	KIAA0528	0.7	EPHX3	0.79

NIPSNAP3A	0.48	CCAR1	0.57	RPS25	0.64	ZNRD1	0.7	IFIH1	0.79

FKBP5	0.48	SMARCC1	0.57	BTAF1	0.64	TUT1	0.7	DIP2A	0.79

TP53BP2	0.48	C9orf40	0.57	CHD7	0.64	PRPF4B	0.7	UBA6	0.79

HNRNPCL1	0.48	Q6ZNC3_HUMAN	0.58	MRE11A	0.64	ANKHD1	0.7	CYP2A7	0.79

BARD1	0.49	JHDM1D	0.58	DEDD	0.64	HHEX	0.7	RAD17	0.8

ASF1A	0.49	LOC100129434	0.58	RPS10	0.65	ZNF493	0.7	ANP32E	0.8

LOC100289152	0.49	VWA5A	0.58	MORN3	0.65	AGTR2	0.7	MRPL28	0.81

KDELC2	0.5	CASP3	0.59	PCDH12	0.65	C14orf118	0.7	LOC284080	0.81

UHRF1	0.5	CBL	0.59	RPS25	0.65	SLMO1	0.7	TRIM52	0.81

NDC80	0.5	N4BP2L2	0.59	RAB11FIP1	0.65	PPIL3	0.7	C21orf45	0.81

KIF2A	0.51	CLK1	0.59	ELMOD3	0.66	C14orf135	0.7	SF4	0.81

FUBP1	0.51	HMGN1	0.59	DPY19L2P2	0.66	WFIKKN1	0.7	BAT2L2	0.82

SRPK1	0.51	CEP110	0.59	LARS2	0.66	BAZ1B	0.71	ZNF646	0.82

FAM111A	0.51	TIA1	0.6	LMBRD2	0.66	SMAD1	0.72	LOC643763	0.82

HNRNPA1	0.52	PHIP	0.6	RAF1	0.66	MRPS25	0.72	ZNF665	0.82

PNN	0.52	RAD51AP1	0.6	PTGES3	0.66	SFRS2IP	0.72	TNFAIP8L2	0.82

CENPK	0.52	PHF3	0.6	ATF7IP	0.66	SFRS3	0.72	LRDD	0.82

TRMT5	0.52	CRY1	0.6	OR1J1	0.66	MIS12	0.72	AKAP2	0.83

POLR2A	0.52	LOC729348	0.6	NIPBL	0.66	PCSK7	0.73	ZNF451	0.83

MSL2	0.52	TBP	0.6	KCTD4	0.66	CASC3	0.73	CAPN11	0.83

BAZ1A	0.53	MLL	0.61	LOC729687	0.66	DOK1	0.73	SCXA	0.83

PRDM10	0.53	TMEM44	0.61	DBF4	0.66	XPO6	0.73	CNO	0.83

RPSAP58	0.53	SFRS12	0.61	MSH3	0.66	RPL19	0.73	ZNF691	0.84

HNRNPA2B1	0.54	SFRS13A	0.61	NUP153	0.66	RALA	0.73	SDAD1	0.84

HNRPDL	0.54	FYTTD1	0.61	DNAH11	0.67	FAU	0.74	LOC284513	0.85

TMPRSS3	0.54	KIF11	0.61	MTA2	0.67	SAFB2	0.74	ANKRD49	0.85

SIKE1	0.54	APPL1	0.62	RBM15	0.67	TRIM77	0.74	TAF3	0.85

POLM	0.54	C7orf68	0.62	USE1	0.67	MTMR15	0.74	GTF2H3	0.86

ZNF295	0.55	PHF10	0.62	RBMXL1	0.67	CNOT8	0.74	GLYR1	0.86

TSPYL4	0.55	LOC728643	0.62	ANP32B	0.67	ANKRD62	0.74	EFR3A	0.86

GUSBP1	0.55	CTDSPL2	0.62	BCL11A	0.67	FUBP1	0.74	LOC100131107	0.87

SFPQ	0.55	LOC729348	0.62	WHSC1	0.67	PPWD1	0.75	ATXN7L2	0.87

LRRCC1	0.55	AGAP5	0.63	MOBKL2A	0.67	IL18R1	0.75	TDG	0.87

RIF1	0.55	SDHAP2	0.63	PRKDC	0.68	SDHAF2	0.75	LOC400406	0.88

HSF1	0.55	XP_942858.1	0.63	PDSS1	0.68	BRAP	0.75	EDA	0.89

CPSF6	0.55	ZRANB2	0.63	CDC40	0.68	FBXO33	0.75	RXRG	0.89

LOC100289563	0.55	OPA1	0.63	LOC646864	0.68	HLTF	0.75	IL17RE	0.89

ZNF117	0.55	GUSB	0.63	ACTR2	0.68	NXF1	0.76	IBSP	0.9

ZNF91	0.56	REL	0.63	DMTF1	0.68	UGT2B15	0.76		

GDF15	9.12	ASAH1	2.09	ART3	1.53	POLR2F	1.22	SOX30	1.13

SYTL2	5.05	B4GALT5	2.05	XP_930957.1	1.51	FAM65C	1.22	ERMAP	1.12

NPAS2	3.97	TSPAN3	2.02	PHPT1	1.51	SSX3	1.22	DHRS7B	1.12

ITGB5	3.89	RNF182	1.95	COX6A1	1.49	SYNM	1.22	CAMKK1	1.12

CPM	3.53	MTFMT	1.95	PHLDA3	1.48	HSF4	1.21	LY6G5C	1.11

DUSP4	3.25	OPN1MW2	1.9	SORT1	1.46	SNORD83A	1.21	KIAA1310	1.11

CD9	3.21	LGI3	1.9	ACADS	1.46	RPL10A	1.2	KRTAP10-8	1.09

KIFC3	3.15	BMPR1B	1.84	LOC158434	1.46	GLT8D2	1.2	OR5R1	1.09

UGT2B28	2.86	C2orf82	1.8	SSU72	1.42	DNAH8	1.18	CACNA1F	1.15

ABCB6	2.83	PSMD8	1.78	AGPAT2	1.4	SLC6A8	1.18	AKT2	1.14

GPRC5B	2.82	TIMM50	1.71	KANK2	1.38	LOC100130472	1.17	ZFYVE20	1.14

BAIAP2L1	2.72	GABBR1	1.7	FN1	1.37	CCK	1.17	MOCS1	1.13

CLDN9	2.59	SYAP1	1.7	ERICH1	1.37	GPR113	1.16	SOX30	1.13

FTHL3	2.58	MRPS12	1.69	LDB3	1.37	EFHA2	1.16	ERMAP	1.12

FTH1	2.49	HOXA1	1.62	TPPP	1.36	PDE4C	1.16	DHRS7B	1.12

IGSF3	2.48	PSMD8	1.62	MCF2L	1.36	PTK2	1.15	CAMKK1	1.12

FTH1	2.41	PRDM13	1.61	C19orf63	1.35	POM121L1P	1.15	LY6G5C	1.11

MRAP2	2.37	TMEM147	1.6	MAGEB17	1.35	VSIG1	1.15	KIAA1310	1.11

GABRA1	2.31	GPI	1.59	ELF3	1.32	NEDD9	1.15	KRTAP10-8	1.09

HOXB7	2.22	RNF113A	1.59	ERMP1	1.31	PGPEP1	1.15	OR5R1	1.09

TSKU	2.15	USP3	1.57	WBSCR17	1.3	CACNA1F	1.15		

C10orf125	2.14	LOC730144	1.57	GBA2	1.3	AKT2	1.14		

AVPI1	2.13	ENTPD6	1.56	CCDC27	1.25	ZFYVE20	1.14		

PYGB	2.09	PHLDA3	1.53	DENND5B	1.25	MOCS1	1.13		

Gene were ordered based on Fold change (Fc)

Nevertheless, three genes: Growth Differentiation Factor 15 (GDF-15), CD9 and Integrin B5 (ITGB5), known to play an important role in cell movement and adhesion, ranked at the top of those up-regulated in permissive cell lines (Table 3).

In addition, genes involved in IRF (Interferon Regulatory Factor) activation by cytosolic pattern recognition receptors and in RNA polymerase II activation complex, were found to be down regulated in permissive cell lines. Those genes included Interferon Induced with Helicase C domain 1 (IFIHI), RNA Polymerase II (POL II), and Reticuloendotheliosis viral oncogen homolog (v-REL) which are also involved in Interleukin 12 (IL-12) signaling and macrophage activation (Table 3).

**Figure 3:**
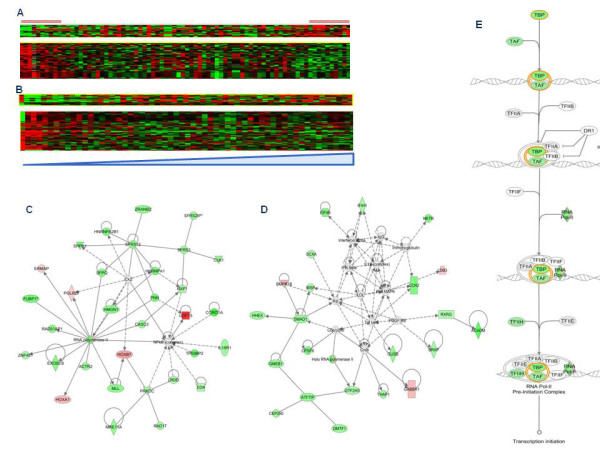
**Heat map based on genes derived from Student T test (p < 0.01) between permissive and not permissive cell lines using in house database and external database**. A, B) Heat maps based on 371 genes derived from Student's T test: comparison between the heat map obtained using our dataset (A) and an independent dataset derived from Genomics and bioinformatics groups, LMP, CCR, National Cancer Institute, Bethesda, MD (B). IPA based on the 317 differentially expressed genes (derived from Student's T test p < 0.01) with functional annotations, constructed a primary network centered on NF-KB signaling (C) and a second ranking network centered on TGF-β and Interferon-α/β signaling (D) where genes up-regulated or down-regulated in higher permissive cell lines are respectively indicated in red and green. E) A canonical pathway analysis related to assembly of the RNA polymerase II complex also showed a down-regulation of this pathway in higher permissive cell lines.

The same 371 genes served as a platform to generate a heat map based on archival NCI-60 cell lines (59 out of the 74 cell lines considered in our study) transcriptional data derived from Genomics and bioinformatics groups, LMP, CCR, National Cancer Institute, Bethesda, MD (http://discover.nci.nih.gov/cellminer/loadDownload.do. This independent dataset demonstrated a comparable pattern of gene expression (Figure 3A, B). Thus, it is likely that the transcriptional profiling obtained in our cell lines is representative and confirmatory of the transcriptional profile of NCI-60 panel cell lines obtained in other experimental conditions. Based on the 317 differentially expressed genes with functional annotations, Ingenuity Pathways Analysis (IPA) constructed a primary network centered on NF-kB signaling (Figure 3C) and a second ranking network centered on TGF-β and Interferon-α/β signaling (Figure 3D). In either case, the majority of transcripts were down-regulated in permissive cell lines as it could have been predicted in teleological terms. In fact, expression of v-REL, IFIHI and POL II and other genes involved in IRF 3/7 signaling and activation of innate immunity were down-regulated in permissive cell lines while few transcripts that were up regulated such as GDF-15 had known inhibitory effects on innate immune function. Similarly, the canonical pathway related to assembly of the RNA polymerase II complex was predominantly down-regulated in permissive cell lines (Figure 3E). Several other statistical approaches to identify predictors of permissivity yielded similar results.

## Conclusions

The Ruc-GFP carrying vaccinia virus strain GLV-1h68 is a leading candidate for oncolytic therapy [27].

Based on GFP expression, we observed that permissivity to VACV infection during the first 20 hours is heterogeneous among cell lines but highly reproducible within each cell line. The tissue origin of each cell line does not influence the permissivity to infection with the exception of B cell derivation. Moreover, permissivity correlates between two VACV constructs and between them and VSV suggesting that a common cancer cell phenotype may influence the replication of these viruses.

Transcriptional profiling did not identify clear correlates of permissivity contrary to the experience of others in different viral systems: for instance, transcriptional profiling proved useful in the identification of platelet-derived growth factor receptor as a receptor of adeno-associated virus type 5 (AAV5) cell entry [28]. In particular, expression profiling of ovarian cancer cell lines resistant or susceptible to viral oncolysis, suggested that the epithelial phenotype of ovarian cancer represents a barrier to infection by adenoviruses [29]. Accessibility to viral receptors was critically linked to the depolarization and the loss of tight and adherence junctions both hallmarks of Epithelial-to-Mesenchymal Transition (EMT) as showed by higher expression of adherens proteins (E-cadherin), tight junction (occludine) and epithelial marker (EPCAM) in resistant cells. Conversely, susceptible cells predominantly expressed markers that are characteristic for mesenchymal cells (vimentin, collagen IV and N-cadherin). Furthermore, expressions of CD44, considered a marker of mesenchymal stem cells, and p120 catenin, were found to be higher in more permissive cell lines. This difference, apparently explains why oncolytic virotherapy works well in xenograft models but very poorly in patients because the tight junctions of epithelial cells shield the adenovirus receptor. When primary tumors are cultured, apparently only the cells with some mesenchymal characteristics reproduce in culture and then tend to further progress through the EMT.

Interestingly, our results showed a similar behaviour for VACV infection confirming that ovarian cell lines less permissive to VACV infection expressed higher level of mesenchymal molecules and lower expression of adherens and tight junction proteins (Additional File 2C). However this phenomena was not observed in cancer cell lines derived from other tissues suggesting that this observation is specific to ovarian cancer and cannot be generalized (Additional File 2A, B).

Class comparison between high and low permissivity cell lines identified over expression of integrins and tetraspanins involved in cellular mobility and adhesion such as ITGB5 and CD9 in highly permissive cell lines. The association between integrins and tetraspanins is common within cells [30] and it has been implicated in adenoviral replication and internalization [31,32] allowing Adenoviral replication even in cell lines lacking adenovirus receptor [33]. CD9 has been also linked to replication of feline immunodeficiency virus [34] and canine distemper virus infections [35]. Thus, it is possible that these two molecules may play a general facilitator role in viral infection for different viruses including VACV and VSV.

In contrast to the upregulation of integrins and tetraspanins, highly permissive cell lines demonstrated constitutive suppression of endogenous innate immune mechanisms regulated around IRF, NF-kB and Interferon (IFN) signaling as reveled by upregulation of GDF-15 (also known as Macrophage Inhibitory Factor I-MIC1) and down regulation of v-REL, IFIHI and POL II and other genes involved in IRF3/7 dependent signaling and IL-12 production by macrophages [36]. The down regulation of the expression of c-Rel, IFIHI and POL2R observed in VACV higher permissive cell lines supports a role played by the host's immune response in limiting vaccinia virus replication, and consequently the effectiveness of oncolytic therapy. Endogenous activation of innate immunity, in cancer cells appears to hamper viral replication in other viral models [19] suggesting that this may be a potential cancer-specific mechanism modulating the effectiveness of oncolytic therapy.

Although this analysis provided some potentially useful insights about potential factors relevant to VACV and VSV replication in cancer cells, the signatures identified could not predict intermediate cancer cells behavior.

We, therefore, conclude that transcriptional profiling is unlikely to provide conclusive information about cell lines permissivity to VACV infection even when tightly controlled experimental conditions are applied. Perhaps higher resolution genomics, functional genomics, epigenetics or protein based techniques may be required for identification of such factors. Nevertheless, the information obtained here may contribute a road map for the interpretation of future studies in which a multi-modality approach will be adopted to address the question of cancer cell lines permissivity to infection.

## Competing interests

The authors declare that they have no competing interests.

## Authors' contributions

MLA designed the study, performed RNA and DNA extraction, RT-PCR screening, infection of cell lines, plaque assay, FACS and microarray experiments with statistical analysis and wrote the manuscript. AW performed infection of cell lines, FACS and microarray experiments with statistical analysis. ZY and JR performed plaque forming assay and expansion of VACV WR strain. SA performed HLA typing of cell lines dataset. PAA, DFS, NGC and AAS designed the study and analyzed the data. ZP, RR, GDP, DB, LU, FR analyzed the data. NR provided VACV-WR strain and analyzed the data. EW performed microarray experiments and statistical analysis. FMM designed the study, followed all the experimental procedure, contributed to new reagents/analytic tools and drafted the manuscript.

All authors gave final approval for the manuscript to be published.

## Pre-publication history

The pre-publication history for this paper can be accessed here:

http://www.biomedcentral.com/1471-2407/11/451/prepub

## Supplementary Material

Additional file 1 **Permissivity of cells lines infected with differential vaccinia strains or with VSV virus and cultured in appropriate cell culture media**. A) Higher and less permissive cell lines infected in parallel with GLV-1h68 or VACV-WR and with GLV-1h68 or VSV. After infection, GFP expression was evaluated by FACS analysis.B) Plaque forming assay of Panc1, NCI-H1299, HT-29 and A549 cell lines cultured in cell specific culture media and infected with GLV-1h68 for 20 hrs. Click here for file

Additional file 2 **Expression hallmarks of Epithelial-to-Mesenchymal-Transition (EMT)**. A) Gene expression of adherens and tight junction transcripts (light blue) and mesenchymal transcripts (purple) occurring in lowest permissive cell lines (n = 20) and highest permissive cell lines (n = 20). Data were based on microarray value. B, C) Self organization of adherens-tight junction (light blue) and mesenchymal transcripts (purple) in 74 cells lines (A) and in ovarian cancer cell lines (B) prior VACV infection. Cells were ranked according infectivity index. Click here for file
