# Supplementary figures and images for: Permissivity of the NCI-60 cancer cell lines to oncolytic Vaccinia Virus GLV-1h68
Source: BMC Cancer. 2011 Oct 19;11:451. doi: 10.1186/1471-2407-11-451 (PMC3213037; doi:10.1186/1471-2407-11-451)

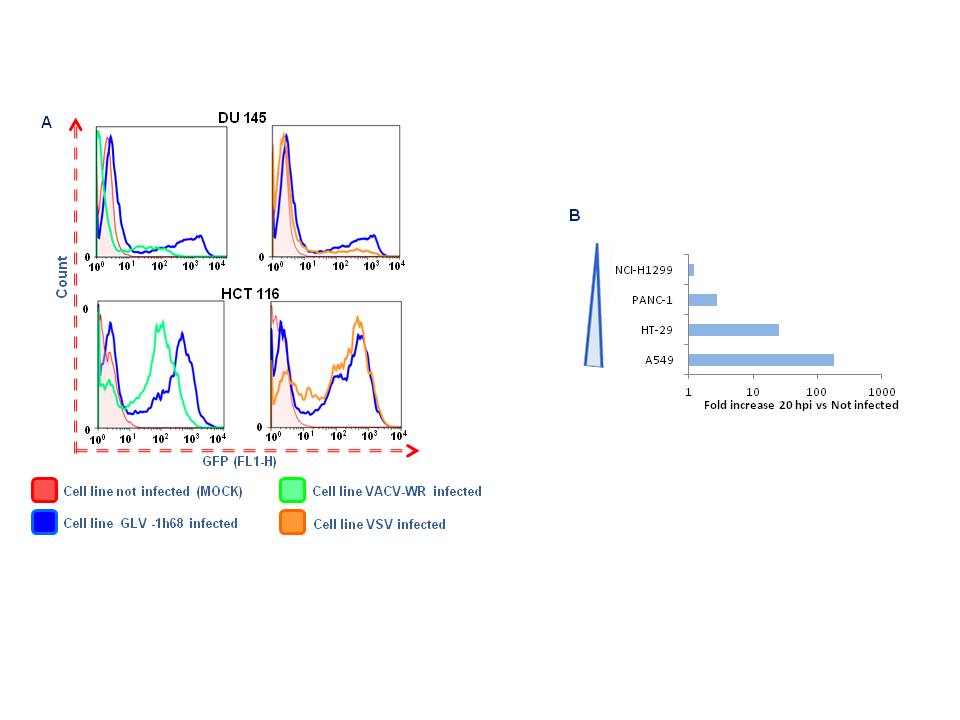

Supplement: Additional file 1 — Permissivity of cells lines infected with differential vaccinia strains or with VSV virus and cultured in appropriate cell culture media. A) Higher and less permissive cell lines infected in parallel with GLV-1h68 or VACV-WR and with GLV-1h68 or VSV. After infection, GFP expression was evaluated by FACS analysis.B) Plaque forming assay of Panc1, NCI-H1299, HT-29 and A549 cell lines cultured in cell specific culture media and infected with GLV-1h68 for 20 hrs. [file 1471-2407-11-451-S1.TIFF]

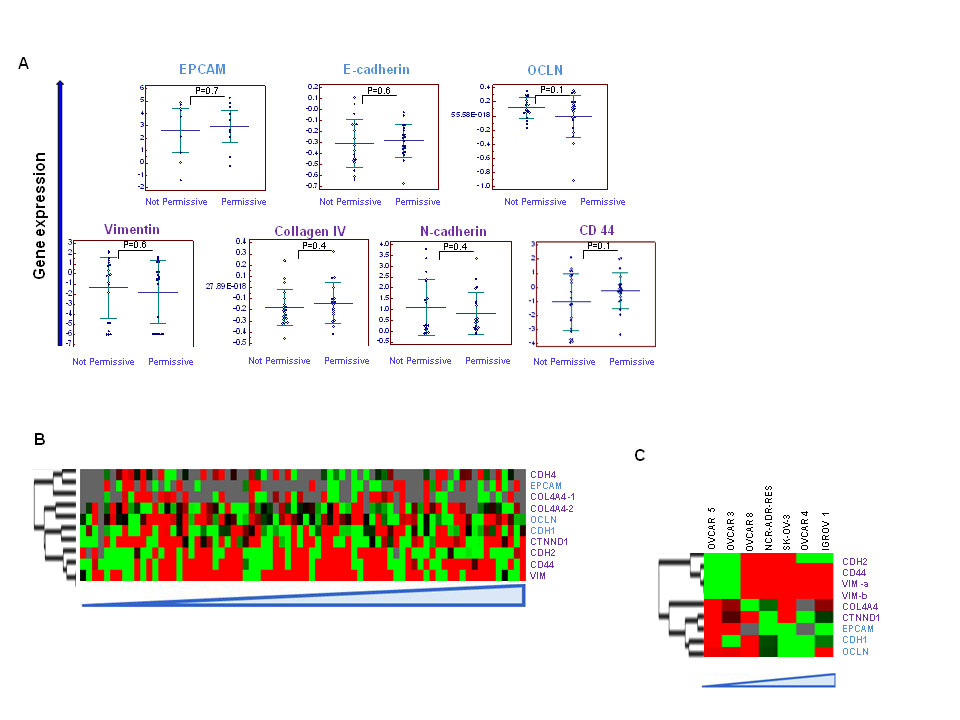

Supplement: Additional file 2 — Expression hallmarks of Epithelial-to-Mesenchymal-Transition (EMT). A) Gene expression of adherens and tight junction transcripts (light blue) and mesenchymal transcripts (purple) occurring in lowest permissive cell lines (n = 20) and highest permissive cell lines (n = 20). Data were based on microarray value. B, C) Self organization of adherens-tight junction (light blue) and mesenchymal transcripts (purple) in 74 cells lines (A) and in ovarian cancer cell lines (B) prior VACV infection. Cells were ranked according infectivity index. [file 1471-2407-11-451-S2.TIFF]
